# Case Report: Challenges of insulin and sulfonylurea dosing in an extremely premature infant for the management of KCNJ11-associated neonatal diabetes mellitus

**DOI:** 10.3389/fped.2025.1456818

**Published:** 2025-04-16

**Authors:** Ishan Perera, Brooke Jensen, Hirenkumar Patel, Melissa Garganta

**Affiliations:** ^1^ Center for Simulation and Technology, Edward Via College of Osteopathic Medicine, Blacksburg, VA, United States; ^2^ Department of Pediatrics, Carilion Roanoke Memorial Hospital, Roanoke, VA, United States; ^3^ Department of Pediatric Endocrinology, Carilion Roanoke Memorial Hospital, Roanoke, VA, United States

**Keywords:** neonatal diabetes mellitus, KCNJ11 mutations, insulin, neonatal endocrinology, genetic testing, NICU, preterm, sulfonylurea

## Abstract

**Introduction:**

Neonatal diabetes mellitus (NDM) is a rare disease, the prevalence of which is 1 in 90,000–160,000 live births, with 31% of all preterm diagnoses linked to monogenic causes. NDM is differentiated into transient, permanent, and syndromic NDM. Furthermore, 40% of patients diagnosed with NDM are responsive to oral sulfonylureas (SUs) due to expressed mutations of the ABCC8 or KCNJ11 genes coding for adenosine triphosphate-sensitive potassium channel (KATP) subunits in pancreatic beta (β) cells. SUs bind to the sulfonylurea receptor 1 subunit, closing the KATP channel and increasing insulin secretion. Although SUs remain the mainstay of NDM treatment, these medications are traditionally only dosed and approved for hyperglycemic control in adults. Current treatment regimens suggest a high dose, 1 mg/kg/day, for patients with KCNJ11 neonatal diabetes.

**Case description:**

Our male neonate was born at 27 weeks and 1,020 g via emergency cesarean section due to complete placenta previa and maternal hemorrhage following perinatal betamethasone administration. Neonatal resuscitation was required. During resuscitation, the patient was intubated and found to be hyperglycemic. He was subsequently started on Regular Humulin at 0.1 units/kg/dose subcutaneously (SQ) and increased to 0.2 units/kg/dose SQ before transfer to our facility on day of life (DOL) 19. In our neonatal intensive care unit, the patient was transitioned to an insulin drip due to difficulty obtaining and administering the appropriate, but minuscule, SQ insulin doses. Genetic testing was positive for a pathogenic variant c.679G>A (p.Glu227Lys) in KCNJ11; therefore, glyburide was started at 0.1 mg/kg/dose twice a day on DOL 54, resulting in euglycemia. Hyperglycemia recurred after an initial attempt to wean; however, a subsequent wean on DOL 70 was successful in maintaining euglycemia without insulin or glyburide for 48 h prior to discharge on DOL 78.

**Discussion:**

This case report describes the unique and complicated clinical course of a premature neonate with an initially undifferentiated class of NDM requiring a microdose-based approach, i.e., a fraction of a regular dose, to both insulin and SU-based management. This report offers a concise recount of the applied diagnostic and therapeutic procedures while suggesting a decision tree for future NDM management in neonates.

## 1 Introduction

Neonatal diabetes mellitus (NDM) or persistent hyperglycemia within the first 6–12 months of life, affects approximately 1 in 90,000–160,000 live births, with 31% of all preterm disease linked to monogenic etiologies (1–3). This disease is differentiated into transient, permanent, and syndromic NDM (1). Moreover, 40% of patients diagnosed with NDM are responsive to oral sulfonylureas (SUs) due to expressed mutations of the ABCC8 or KCNJ11 genes coding for adenosine triphosphate (ATP)-sensitive potassium channel (K_ATP_) subunits in pancreatic beta (β) cells. ABCC8 codes for the regulatory subunit sulfonylurea receptor 1 (SUR1), which is a modulatory subunit that binds SU and MgADP, contributing to the closure of the K_ATP_ channel and increasing insulin secretion from pancreatic β-cells (4–6), whereas KCNJ11 codes for Kir6.2, which composes adenosine triphosphate-sensitive potassium channel pores (ATP-sensitive K-channel pores) in pancreatic β-cells (1–4, 7, 8). These ATP-sensitive K-channel pores bind ATP, which promotes channel closure and the exocytosis of insulin-containing granules (4, 9). Although SUs remain the mainstay of NDM treatment, these medications are traditionally only dosed and approved for hyperglycemic control in adults (2, 10). Current treatment regimens suggest starting with subcutaneous (SQ) insulin and then transitioning to high-dose SU, 1 mg/kg/day as most appropriate for patients with KCNJ11 neonatal diabetes (10–13). This case report describes the difficult clinical course of a premature neonate with an initially undifferentiated class of NDM requiring a microdose-based approach, i.e., a fraction of a regular dose, to both insulin and SU-based management for oral feed escalation.

## 2 Case description

At the transferring hospital, a 27-week-old male infant with a birth weight of 1,020 g was delivered via emergency cesarean section due to complete placenta previa with maternal hemorrhage following perinatal betamethasone administration. At delivery, the patient required resuscitation including intubation with Apgar scores of 3, 6, and 7 at 1, 5, and 10 min, respectively. During postnatal stabilization, the patient was identified to be hyperglycemic. Following stabilization, he was weaned to nasal continuous positive airway pressure (nCPAP) and loaded with 20 mg/kg of caffeine before transitioning to 5 mg/kg maintenance caffeine for risk of apnea of prematurity. He was transfused with one unit of packed red blood cells (pRBCs) for neonatal anemia secondary to maternal blood loss and started on empiric intravenous (IV) ampicillin and gentamicin due to infection risk. Antibiotics were discontinued at 48 h following completion of the sepsis rule-out.

On day of life (DOL) 1, the patient was hyperglycemic with levels between 250 and 350 mg/dl (13.9–19.4 mmol/L). Secondary to his hyperglycemia, his IV dextrose-containing fluids were reduced from a glucose infusion rate (GIR) of 5.6 mg/kg/min to a GIR of 2.8 mg/kg/min and he was started on Regular Humulin at 0.1 units/kg/dose SQ. Trophic feeds were started via a gavage tube, increasing the total fluids to 150 cc/kg/day due to hypernatremia of 152 mmol/L. On DOL 10, in the setting of recurrent hyperglycemia and potential for sepsis, blood cultures and a lumbar puncture (LP) were performed. The LP was reassuring but the blood culture was positive for coagulase-negative *Staphylococcus* so a 10-day course of vancomycin was started. On DOLs 12–14, feeds were fortified to 26 kcal/oz of the mother's own breast milk (MBM)/donor breast milk (DBM) and dextrose fluids were changed to 0.45% normal saline with total fluids reaching 160 cc/kg/day. An echocardiogram (ECHO) demonstrated a patent ductus arteriosus (PDA), patent foramen ovale (PFO), and dilated pulmonary veins. The PDA was treated with indomethacin and excess fluid was controlled with furosemide, chlorothiazide, and spironolactone. Due to hyperglycemic glucometer readings of 229–266 mg/dl (12.7–14.8 mmol/L) and glucosuria without ketones, his insulin dose was increased to 0.2 units/kg/dose SQ.

All the aforementioned events occurred before the neonate's transfer to our facility. He arrived on DOL 19 at 29 weeks and 5 days corrected gestational age (CGA) for persistent hyperglycemia. Additional history was obtained from interviews with the parents following the transfer. The interview revealed that the patient's father was also born preterm at 25 weeks gestation, was diagnosed with NDM, and required a 7-month admission to the neonatal intensive care unit (NICU). His parents report a strong family history of diabetes mellitus (DM) and his father has a current diagnosis of type 1 DM (diagnosed at 12 years old).

On arrival, the patient was breathing comfortably on nCPAP with a grade 2–3/6 holosystolic murmur and no acute findings. Initial labs demonstrated a glucose greater than 200 mg/dl (11.1 mmol/L) and hyponatremia. On DOL 22, due to difficulty maintaining euglycemia and the potential for overdosing on SQ insulin, the patient was started on an insulin drip of 0.007 units/kg/h, titrated to maintain blood glucose >80 mg/dl (4.4 mmol/L) and <200 mg/dl (11.1 mmol/L). To optimize this regimen, he began continuous feeds via a gavage tube at 26 kcal/oz supplemented with medium-chain triglycerides (MCTs). Each day, his continuous feed rate was titrated up by 0.5 ml/h to a goal of 9.5 ml/h. On DOL 40, nCPAP was weaned to room air. Five days later, on DOL 45 or 33 weeks 3 days CGA, his genetic testing revealed a heterozygous pathogenic variant, c.679G>A (p.Glu227Lys) in KCNJ11. Concurrent testing of his father revealed an identical heterozygous pathogenic variant. We began a twice-a-day (BID) oral feed trial (5 ml) on DOL 47 and condensed his oral feeds to occur every 4 h on DOL 49. He continued to receive insulin at 0.007 units/kg/h while awaiting an appropriately concentrated glyburide solution from a nearby compounding pharmacy. On DOL 54, he was started on a 0.1 mg/kg BID glyburide suspension and his insulin drip was weaned (0.004 units/kg/h for 1 day then discontinued) with an initial blood glucose of 102 mg/dl (5.7 mmol/L). The glyburide alone maintained the patient’s euglycemia between 100 and 200 mg/dl (5.6 and 11.1 mmol/L, respectively) and thus, a wean was attempted starting on DOL 55. Given the patient's small size, there was difficulty measuring doses smaller than 0.1 mg/kg of glyburide. A smaller concentration (a microdose) of glyburide was obtained from a local compounding pharmacy, allowing the wean to proceed. Over the next 8 days, the patient was slowly weaned off the glyburide, decreasing the dose by an average of 0.01 mg/kg/day. A transition from scheduled feeding to feeding per os ad lib (POAL) without glyburide was attempted on DOL 63 due to the minuscule amount of glyburide being administered; however, this resulted in significant hyperglycemia greater than 200 mg/dl or 11.1 mmol/L, so glyburide was restarted. Following a week of euglycemia, another attempt was made to wean the glyburide by decreasing 0.01 mg/kg/day. The patient remained euglycemic for an additional 48 h off glyburide before discharge. He was discharged on DOL 78 at 38 weeks and 1 day CGA with instructions for his parents to begin at-home glucose monitoring. Endocrinology follow-up for close monitoring was recommended due to this patient's increased risk of diabetes in late adolescence or early adulthood. Developmental screenings and neurology referrals were left to the discretion of the patient's primary care physician as the pathogenic variant c.679G>A (p.Glu227Lys) in KCNJ11 is primarily associated with maturity-onset diabetes of the young (MODY) and is low risk for developmental delay, epilepsy, and neonatal diabetes (DEND) syndrome.

## 3 Discussion

In this article, a premature neonate presented to our facility for persistent hyperglycemia and was ultimately discovered to have NDM and be positive for a heterozygous pathogenic variant, c.679G>A (p.Glu227Lys), in KCNJ11. Following the traumatic delivery of this neonate, his NICU course was complicated by several factors including neonatal respiratory distress syndrome (requiring positive pressure ventilation), anemia (requiring PRBCs), electrolyte abnormalities (resolved with feeds and fluid management), cardiac concerns (managed with an indomethacin course), infection (treated with antibiotics), and persistent hyperglycemia (requiring insulin and glyburide). The significant duration of the patient's hyperglycemia secondary to NDM highlights the importance of early genetic testing for this disease as the switch from insulin to SU was ultimately only recommended due to the insight provided by genetic testing. Eventual SU wean confirms the transient classification of this patient's NDM and warrants consistent follow-up for possible re-emergence of diabetes in adolescence or adulthood (MODY).

NDM is associated with the overexpression of genes at the 6q24 locus and mutations in genes encoding either subunit of the K_ATP_ channel. KCNJ11 and ABCC8 mutations are the most commonly implicated in this disease (1–3, 7, 8). The K_ATP_ channel utilizes ATP and MgADP as signaling molecules in response to elevated blood glucose levels; the rise in ATP concentration secondary to glycolysis leads to the closure of this channel, and thus membrane depolarization, allowing calcium ions to enter the β cell and facilitate the release of insulin (2, 4, 5, 7, 9, 14). KCNJ11 mutation typically impairs the coding for ATP-binding pocket receptors and is associated with improved patient outcomes and response to SU-based therapy (1–3, 5, 7, 8, 12, 15). Our patient's single nucleotide missense mutation increased resistance of the K_ATP_ channel to MgATP-induced inhibition, leading to a mostly open channel (15, 16). Gain-of-function variants are present in approximately 35% of patients with NDM (15), leading to a constantly open K_ATP_ channel preventing depolarization and the release of insulin (17).

Diagnosis of NDM is difficult in the neonatal period as there are often multiple confounders at play. These confounders increase in premature patients secondary to increased frequency of perinatal steroid administration, early glucose administration, and increased rates of sepsis (1, 12). Considering these confounding variables, NDM linked to KCNJ11 or ABCC8 is typically diagnosed at a median age of 9.6 weeks, with diagnoses before 4 weeks old in only one-third of cases and two-thirds of cases diagnosed between 1 and 6 months old (1, 2). Our 27-week premature infant was diagnosed at 6 weeks and 3 days or DOL 45 despite multiple confounders. The literature supports genetic testing in the setting of hyperglycemia early in the diagnostic course (12), sometimes within the first 2–3 weeks of life (1). NDM should be high on providers’ differentials for a newborn with persistent hyperglycemia, as a faster insulin-to-sulfonylurea transition is preferred (1–3, 8, 11, 13, 14). Importantly, the gene has been noted to have varying penetrance and a potential for *de novo* occurrence (9, 15). It is important to ensure patients with the KCNJ11 gene mutations receive appropriate follow-up appointments and guidance due to the potential for complications (3, 8, 11, 12, 18). In addition, considering the father's concomitant positive genetic test and history, it was recommended that he follow up with his endocrinologist for consideration of SU-based therapy. It has been previously demonstrated that intermittent continuous glucose monitoring (iCGM) allows for a safe and affordable transition to home care (17). This patient's specific variant, c.679G>A (p.Glu227Lys) in KCNJ11, has been seen in MODY type 13 and is inherited in an autosomal dominant manner (19). Diabetes relapse typically occurs before 10 years of age (15). Upon recurrence, data supports the use of the MODY probability calculator, which demonstrated >90% specificity in patients with type 1 or 2 diabetes (9). There are currently no reported cases of this variant leading to DEND syndrome; however, for many KCNJ11 mutations, neurology appointments are highly recommended as approximately 25% of patients with KCNJ11 mutations develop DEND syndrome (8, 16). It is theorized that due to the presence of KATP channels in the brain, patients with KCNJ11 mutations exhibit an increased frequency of attention deficit disorder, sleep disruptions, developmental delays, and seizures (18).

Treatment initially favors proper maintenance of caloric intake with insulin supplementation for restoration of normal weight without triggering excessive insulin spikes leading to increased insulin resistance (2, 6). Traditionally, it is recommended to initiate an insulin infusion between 0.02 (20) and 0.05 units/kg/h continuously (21); however, due to our patient's sensitivity to insulin (as demonstrated by the outside hospital), our insulin drip infused at 0.007 units/kg/h continuously to maintain euglycemia of 120–150 (6.7–7.8 mmol/L). In patients of this size, it is important to administer microdoses or fractionated doses for medications impacting the endocrine system. Awaiting genetic confirmation before starting SUs or starting empiric SUs as a trial are both accepted approaches to NDM of undifferentiated etiology (8, 10). It is important to appreciate the narrow therapeutic index of insulin-based treatment in pediatric patients, with an increased caution recommended in premature infants with a drastically smaller dosage requirement due to weight-based dosing suggestions, as episodes of hyper and hypoglycemia are detrimental to early developmental stages (2, 6). Anticipating the potential for hypoglycemic episodes, our care team immediately transitioned the patient to an IV insulin drip, allowing for appropriate adjustments to insulin dosing in response to his small bolus feeds and weight. Glyburide, the SU most commonly used to transition from insulin to glyburide, has a recommended starting dose of 0.1 mg/kg/dose BID (10); however, many reported cases require much more (1, 8, 9). Notably, 44% of type 2 diabetes mellitus (T2DM) patients fail SU treatment after 5 years, while NDM patients were found to maintain a strong response even after 10years of treatment (11, 17). In our case, glyburide was weaned once a euglycemic steady-state was achieved, following a discussion with Pediatric Endocrinology and the family. Although an SU was transiently used in our case, it is important to recognize its potential continued utility. Once euglycemia was achieved, an SU administered at that steady-state dose could be utilized indefinitely in concurrence with glucose monitoring and family education to lower the dose if hyperglycemia were to occur.

**Figure 1:**
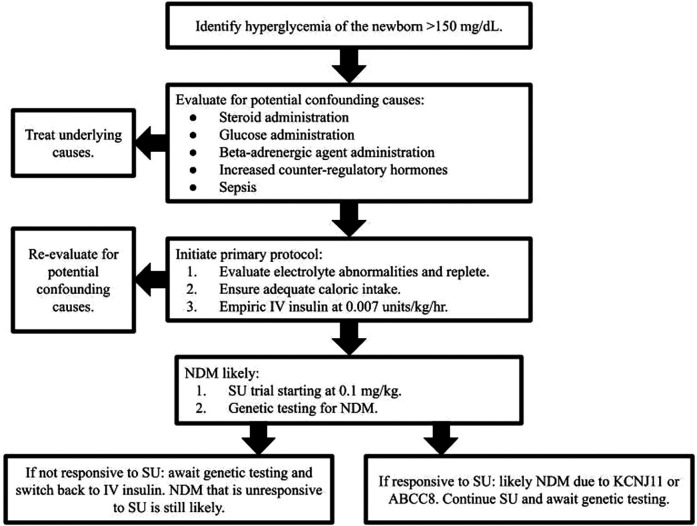
Newborn hyperglycemia decision tree for neonatal diabetes mellitus.

In conclusion, the treatment of NDM, although well documented, does not address the risk of overdose due to SQ insulin administration. Our case suggests foregoing SQ administration for the titration capabilities of an IV insulin drip, with a recommended initial microdose of 0.007 units/kg/h. The total assemblage of medical decision-making from this case is presented in Figure 1. Our team recommends beginning the evaluation of hyperglycemia of the newborn by first identifying confounding variables such as steroids, glucose administration, beta-adrenergic agents, increased counter-regulatory hormones, or sepsis. If these variables do not explain the patient's hyperglycemia, we then suggest a protocol including an evaluation of electrolyte abnormalities followed by repletion, ensuring adequate caloric intake, empirically beginning a microdosed IV insulin drip, and further assessing for a family history of NDM. If NDM is likely, we recommend beginning an SU trial at a 0.1 mg/kg BID microdose and performing genetic testing simultaneously. Close monitoring is recommended if SU administration is performed before the return of genetic testing to ensure hypo- or hyperglycemia is not occurring with a low threshold for switching back to insulin-based therapy if needed. If the SU is unable to be dosed at appropriate levels due to patient size, our group recommends using a compounding pharmacy (if available), to obtain the SU at a more diluted, and therefore measurable, concentration.

## Data Availability

The original contributions presented in the study are included in the article/Supplementary Material, further inquiries can be directed to the corresponding author.
